# A study of post-operative clinico-imaging status and visual outcome after idiopathic macular hole surgery with amniotic membrane plug

**DOI:** 10.22336/rjo.2022.50

**Published:** 2022

**Authors:** Mayank Srivastava, Vinod Kumar Singh, Ankita Singh, Basant Kuma Singh

**Affiliations:** * Department of Ophthalmology, RIO, M.L.N Medical College, Prayagraj, India

**Keywords:** macular hole, hAM, amniotic membrane

## Abstract

**Objective:** The aim of this study was to determine the efficacy of hAM plug in the treatment of idiopathic macular hole and to see its post-operative visual improvement and anatomical apposition.

**Material and methods:** 10 eyes of 10 patients who had idiopathic MH underwent a pars plana vitrectomy (PPV) with the hAM plug implanted in MH. The patients were followed up on 2nd day, 1st week, 3rd week, 6th week and 3rd month.

**Results:** Final anatomical closure of MH was achieved in all the cases. BCVA improved from 0.91±0.11 logMAR to 0.28±0.06 logMAR after 3 months. No adverse event was documented in the specified period.

**Conclusion:** hAM plug is an efficient method to treat and manage idiopathic MH with encouraging results both in terms of anatomical closure and visual acuity gain.

**Abbreviations:** MH = Macular Hole, IOP = Intra Ocular Pressure, ILM = Internal Limiting Membrane, BCVA = Best Corrected Visual Acuity, OCT = Optical Coherence Tomography, LogMAR = Logarithm of Minimum Angle of Resolution, hAM = Human Amniotic Membrane, RPE = Retinal Pigment Epithelium

## Introduction

Macular Hole (MH) is a retinal pathology characterized by a full or partial thickness anatomical opening in fovea centralis. It can result in reduction or loss of central vision, metamorphopsia and central scotoma in the affected eye [**1**].

Usually, the condition is less common and idiopathic in nature, occurring in 1 in 500 patients. It occurs mainly in healthy individuals in their sixth and seventh decade of their life with females showing a higher preponderance, with female to male ratio of 2:1 [**2**]. The most common aetiology of MH is idiopathic, but can also be seen in high myopia and may be due to trauma [**3**]. Several hypotheses for the development of macular hole have been highlighted, among which Vitreous Macular Traction, and the role of Muller cells has been clearly studied [**4**]. Surgical closure of MH is the only treatment modality prevailing so far to treat it. The approach of surgery is to identify and remove vitreotractional forces that can be tangential, antero-posterior or both. MH surgery has evolved over a period of time, procedures including pars plana vitrectomy, posterior vitreous detachment, internal limiting membrane peeling, filling vitreous cavity with gas bubble, post op face down, and inverted flap technique (for relatively large MH) [**5**]. Recently, MH surgery has evolved using human amniotic membrane (hAM). The use of amniotic membrane in ophthalmic surgery is on rise due to its anti-fibrotic, anti-inflammatory, anti-angiogenic and antimicrobial features [**6**]. It is a rich source of biologically active factors, thus promoting healing and acting as active material for wound dressing that also helps in epithelialization. It is transparent and usually lacks any immunogenicity [**7**]. It is already being used for ocular surface reconstruction in nonhealing ulcer, bullous keratopathy, limbal stem cell deficiency, corneal perforation, corneo-scleral melt and glaucoma surgery. Its efficacy is also studied in treatment of choroidal hole due to supra choroidal silicone oil migration [**8**]. Recently, hAM has been advocated for treatment of MH, even one associated with high myopia with good outcome both anatomically and functionally [**9**]. It has showed excellent growth support property in retinal pigment epithelium cells in sub retinal space.

## Material and Methods

The proposed study was carried out at the Regional Institute of Ophthalmology (M.D. Eye Hospital, Dr. Katju Road, Nakhas Kona, Prayagraj) after taking permission from the ethical committee of M.L.N. Medical College, Prayagraj. The patients signed an informed written consent for the surgical procedure, investigations, and use of clinical photographs. Macular Hole was diagnosed by history and clinical examination. Diagnosis was confirmed by slit lamp examination and Optical Coherence Tomography (OCT) (Cirrus HD OCT software version 6.5.0.772; Carl Zeiss Meditec).

The Inclusion criteria were the following: patients of any age group having MH, presence of idiopathic MH, MH with clear media.

The exclusion criteria were the following: any retinal pathology including dystrophy, retinal detachment other than MH, diabetic retinopathy, vascular occlusion, compromised clarity of media, hypertensive retinopathy, any physical or systemic condition contra-indicating prone or head down position.

**Fig. 1:**
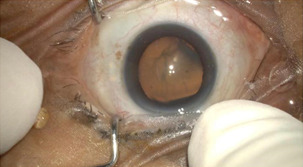
Prepared part

**Fig. 2:**
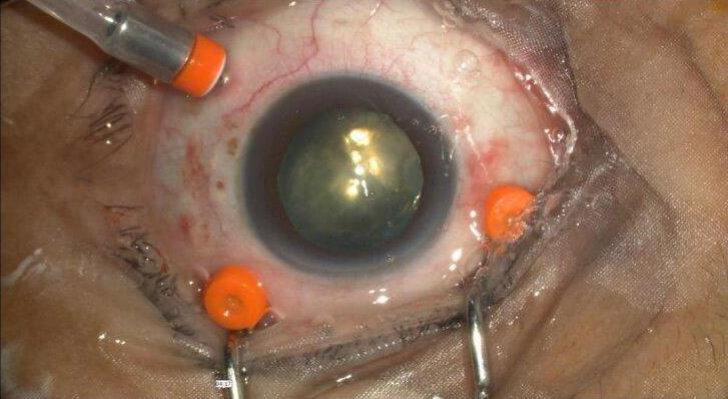
3 port 23 gauze PPV

All the surgeries were performed by a single experienced surgeon. None of the selected patients had undergone any prior posterior segment surgery of the eye. In this study, all the patients underwent the same procedure. 10 eyes of 10 patients underwent 3 port 23 gauze pars plana vitrectomy (**Fig. 1**,**2**).

**Fig. 3:**
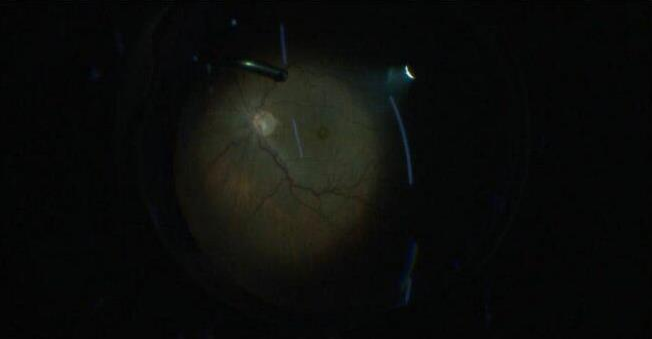
Posterior segment showing MH

**Fig. 4:**
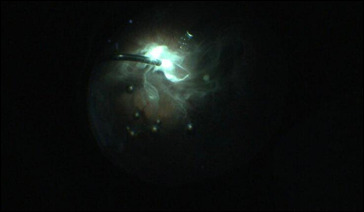
Vitrectomy along with posterior vitreous detachment

**Fig. 5:**
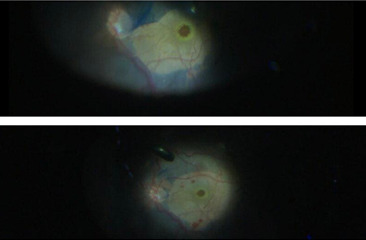
ILM peeling up to arcades

**Fig. 6:**
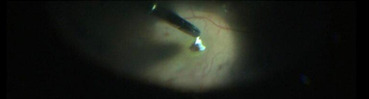
hAM placement into MH

**Fig. 7:**
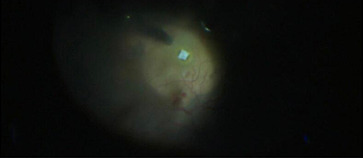
hAM placed in sub RPE space

Vitreous was stained by triamcinolone acetonide, which was available in concentration of 40 mg/ ml, as it helps in better visibility. Posterior vitreous detachment was induced and proper vitreous base shaving was done as seen in **Fig. 4**. Internal Limiting Membrane (ILM) was stained with brilliant blue dye, which was kept in contact for about 3 minutes (**Fig. 3**). ILM peeling was done up to superior and inferior arcades as seen in **Fig. 5**. Then hAM plug was taken. Its dimension was adjusted according to the macular hole size with vitreo-retinal scissors. The hAM plug was then inserted with the help of ILM peeling forceps (**Fig. 6**), through trocar and placed over the hole sticky side facing towards the Retinal Pigment Epithelium (RPE). Margins of plug were undermined into the sub-retinal space placed such that the entire MH was covered with hAM (**Fig. 7**). To prevent the dislocation of plug, visco-elastic was placed over it. At last, after fluid-air exchange, gas-air exchange was done with C3F8 (which was used in its ISO expansile gas i.e. 14%). All the patients were advised to maintain prone position for 14 days.

A fresh piece of amniotic membrane was used in every case. An amniotic membrane was obtained from RIEB, LVPEI, Hyderabad.

The follow up was done on post-op 2nd day, 1st week, 3rd week and 3rd month.

At each follow up visit patients were evaluated for visual acuity (Snellen’s chart), Best corrected visual acuity (BCVA), Intra ocular pressure (IOP), slit lamp examination, fundus examination (direct and indirect ophthalmoscopy) and OCT was done.

**Fig. 8:**
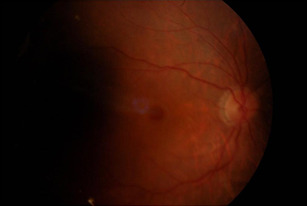
Pre-op fundus picture

**Fig. 9:**
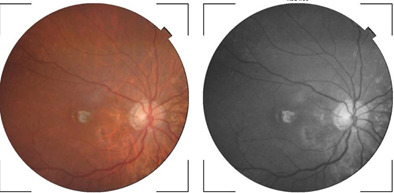
Post-op fundus picture

**Fig. 8** and **9** show an example of one case of pre-op and post-op fundus respectively.

**Fig. 10:**
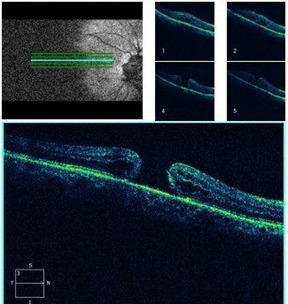
Pre-op OCT

**Fig. 11:**
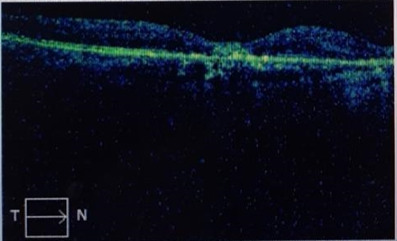
Post-op OCT

**Fig. 10** and **11** show an example of one case of pre-op and post-op OCT respectively.

## Results

We included 10 eyes of 10 patients of idiopathic macular hole (MH). Out of a total of 10 patients, 20% were males and 80% were females. Final anatomical closure of MH was achieved in all the cases.

In our study, none of the 10 eyes of the 10 patients had prior undergone any intervention in the posterior segment. The range of patients’ ages was 50-75 years. The mean average age in our study was 63.2±4.77 years. After 3 months follow up, the mean pre-op BCVA was 0.91±0.11 logMAR and improved to 0.28±0.06 logMAR. No adverse event was documented in the specified time period. 

**Table 1** shows the demographic, pre-op and post-op details of all 10 patients.

**Table 2** shows the mean pre-op and post-op MH size and vision.

**Table 1:** Pre-operative and post-operative demographic data and findings, and results

S. No.	AGE (in years)	SEX	EYE	Lens Status	MH size(minimum diameter inµ)	Baseline BCVA	Final BCVA	Baseline logMAR BCVA	Final logMAR BCVA	Final outcome OCT
1.	67	F	RE	Pseudophakic	973	6/ 60	6/ 18	1	0.4	Closed
2.	70	M	LE	Pseudophakic	391	6/ 36	6/ 9	0.77	0.17	Closed
3.	70	F	RE	Pseudophakic	880	6/ 36	6/ 12	0.77	0.3	Closed
4.	58	F	RE	Pseudophakic	700	6/ 60	6/ 12	1	0.3	Closed
5.	59	F	LE	Pseudophakic	891	6/ 36	6/ 12	0.77	0.3	Closed
6.	59	F	LE	Pseudophakic	806	6/ 60	6/ 12	1	0.3	Closed
7.	64	M	RE	Pseudophakic	944	6/ 60	6/ 12	1	0.3	Closed
8.	61	F	LE	Pseudophakic	987	6/ 36	6/ 9	0.77	0.17	Closed
9.	72	F	LE	Pseudophakic	797	6/ 60	6/ 12	1	0.3	Closed
10.	66	F	RE	Pseudophakic	901	6/ 60	6/ 12	1	0.3	Closed

**Table 2:** Showing mean MH size, and pre-op and post-op BCVA

MH (minimum diameter in µ)	Pre-op BCVA (logMAR)	3 months Post-op BCVA (logMAR)
827±176	0.91±0.11	0.28±0.06

## Discussion

The was prospective and hospital-based, performed with patients having idiopathic macular hole surgery in the Regional Institute of Ophthalmology, M.L.N Medical College, Prayagraj. 10 eyes of 10 patients with idiopathic macular hole (MH) were included. Out of a total of 10 patients, 20% were males and 80% were females. It is known to every ophthalmologist that human amniotic membrane has been used in superficial ocular pathologies for a very long time. But, the use of hAM plug in retinal pathology is a new idea. Though, the use of hAM plug and its role in MH has not been fully understood or thoroughly studied. It has been proposed that hAM helps in the induction of proliferation of RPE and even in their differentiation. Thus, eventually it helps in the closure of MH, as well as in the recovery of the original retinal function. hAM is placed in sub retinal space, as at this point it is in maximum contact with RPE and hence the maximum proliferation and retrieval of function can be achieved. Still, very few studies that used hAM in the surgery of macular hole have been recorded. In 2019, Caporossi T et al. [**6**] carried out a study to access the efficacy of hAM in treating macular hole associated with retinal detachment. They analyzed 10 eyes of 10 patients who had high myopic macular hole. In their study, the mean age was 62.2±9.88 years (range was 49-79 years). Their mean pre-op BCVA was 1.73±0.44 logMAR. They followed up the patients for up to 6 months. They concluded that complete retinal reattachment with MH closure was seen in all the patients. At the end of 6 months, the mean post-op BCVA improved to 0.93±0.23 logMAR.

However, in another study carried out in the same hospital in 2018, Williams GA [**9**] extended this study including fewer patients than in the above-mentioned study, which was performed by Rizzo S and Caporossi T. They enrolled 14 patients who were divided into two groups, of which 8 had recurrent MH and 6 had retinal detachment. In the recurrent MH group, the mean pre-op BCVA was 1.48±0.49 logMAR that ranged from 2 to 0.7 logMAR. They all underwent hAM plug transplantation. At the end of 3 months post-op, BCVA was 0.71±0.37 logMAR. These patients were followed up to 6 months and at the end of 6 months it was 0.48±0.14 logMAR. Complete anatomical closure was seen in all the patients, which was evaluated using OCT. In both studies, patients underwent prior pars plana vitrectomy.

Yadav N et al. [**10**] carried out a retrospective, interventional, comparative case series of 10 patients who underwent hAM plugging for a MH. Seven patients had idiopathic full thickness MHs, 1 patient had traumatic MH, and 1 patient had a MH induced retinal detachment and 1 combined retinal detachment. The control group included 10 cases with similar configuration and duration of MHs but were treated with the inverted peeling of the internal limiting membrane technique. All patients underwent a standard 3 port, 25 gauge transconjunctival pars plana vitrectomy and hAM plug transplantation in the subretinal space under the MH. The anatomic and functional outcomes were assessed at 4 weeks post-surgery. The mean age in the hAM plug group was 62±15.9 years, and in the control group, it was 67.6±4.6 years, respectively. At the 4 week follow up visit, 100% of the cases in the hAM plus group achieved hole closure, whereas 80% of the eyes in the control group were able to achieve hole closure. The mean postoperative VA improved in both groups from the baseline. However, it was not statistically significant.

Ferreira MA [**11**] performed a retrospective chart review that was carried out in five different centers to identify all cases that underwent off-label human amniotic membrane graft for the treatment of large or failed macular holes (MH). 19 eyes of 19 patients were identified and included in the study. The mean age was 66.21±14.96 years and the patients were predominantly females (84%). Out of the total of 19 patients, MH MLD≥650 microns were observed preoperatively in 14 patients. All eyes had successfully closed MH with a single intervention and no recurrences during a mean period of follow-up of 9±3.87 months. The median BCVA in logMAR preoperative was 1.30±0.44 (0.80–2.0), and the median BCVA in logMAR postoperative was 1.0±0.72.

At the same time, Abohussein et al. [**12**] presented a retrospective case series of 14 eyes (14 patients) with macula of retinal detachment. These patients had a macular hole coexistent with peripheral retinal breaks. A human amniotic membrane plug was used to close the macular hole during vitrectomy without ILM peeling. The mean preoperative BCVA (logMAR value) was 1.87±0.31, which improved to 0.67±0.17 at end of 6 months. There were ten females and four males. The mean age was 63.58±5.69 years (ranging from 52 to 71 years). Four patients were phakic and ten patients were pseudophakic. At the 6-month follow-up, all patients showed complete retinal reattachment with macular hole closure.

In our study, none of the 10 eyes of the 10 patients had prior undergone any intervention in the posterior segment of the eye. The range of the patients’ ages was 50-75 years. The mean average age in our study was 63.2±4.77 years. We followed up the patients for 3 months. The mean pre-op BCVA in our study was 0.91±0.11 logMAR. At the end of 6 months, all the patients showed complete anatomical closure, which was evaluated using OCT. The mean post-op BCVA improved to 0.28±0.06 logMAR. We included patients strictly with idiopathic MH, with no any other associated retinal pathology, since it provided a better insight of hAM plug use in the treatment of MH and assessed the required outcome. No post-operative complications were observed in our study.

Moreover, in our study, the final visual outcome in most of the cases was extremely good and this was up to near normal (Snellen’s 6/ 12 to 6/ 9). The achievement was probably possible because our patients had idiopathic MH without any associated retinal pathology. However, the size of MH was almost comparable to the one in other studies.

We were all very much encouraged by the fact that we did not observe any complications in our patients post-operatively. This might be because we were very vigilant during the post-operative period and we personally counselled every patient. However, to have some conclusions, a higher number of patients is needed, as our study included only 10 patients, which was obviously a small series. Simultaneously, we followed patients only for 3 months. Ideally, to comment upon the occurrence of any complications, more time should have been devoted for follow-up. But, we are still following the patients to observe any deterioration of vision or complications if any.

## Conclusion

Based on the observations of our study, we can safely conclude that hAM use in the treatment of MH has promising results and should be explained as an upcoming treatment modality. For better reliability and interpretation of this method of treatment of MH we recommend that this procedure is employed while taking a large sample and following up patients for a larger period of time. This will provide a better insight into the procedure regarding the outcome both in terms of anatomical closure, as well as functional improvement. Further, we can demonstrate complications if any occur during the treatment. 

**Conflict of Interest statement**

The authors state no conflict of interest.

**Informed Consent and Human and Animal Rights statement**

Informed consent has been obtained from all individuals included in this study.

**Authorization for the use of human subjects**

Ethical approval: The research related to human use complies with all the relevant national regulations, institutional policies, is in accordance with the tenets of the Helsinki Declaration, and has been approved by the ethical committee of M.L.N. Medical College, Prayagraj.

**Acknowledgements**

None.

**Sources of Funding**

None.

**Disclosures**

None.
